# Inflammasome pathways in atopic dermatitis: insights into inflammatory mechanisms and therapeutic targets^☆^

**DOI:** 10.1016/j.abd.2025.501136

**Published:** 2025-06-23

**Authors:** Yasmim Álefe Leuzzi Ramos, Anna Julia Pietrobon, Franciane Mouradian Emidio Teixeira, Valeria Aoki, Maria Notomi Sato, Raquel Leão Orfali

**Affiliations:** Department of Dermatology, Laboratório de Investigação Médica em Dermatologia e Imunodeficiências (LIM56), Faculdade de Medicina, Universidade de São Paulo, São Paulo, SP, Brazil

**Keywords:** Cytokines, Dermatitis, atopic, Immunity, innate, Inflammasomes

## Abstract

Atopic dermatitis (AD) is a chronic inflammatory skin disease characterized by a complex interaction between genetic, immunological, and environmental factors. The combination of immune dysregulation and skin barrier dysfunction plays a crucial role in the pathogenesis of the disease. The inflammasome, an important intracellular complex of pattern recognition receptors (PRRs), plays a crucial role in the cutaneous inflammatory response, activating caspase-1 and promoting the release of pro-inflammatory cytokines such as interleukin (IL)-1β and IL-18. The role of inflammasome components in regulating the inflammatory response in AD highlights how the activation of these complexes exacerbates inflammation and contributes to the worsening of the disease and tissue damage. The review included observational and experimental studies investigating inflammasome activation in AD and other inflammatory skin diseases. The main mechanisms of inflammasome activation and their impact on the inflammatory environment and skin barrier integrity were discussed. Understanding the role of the inflammasome in AD is essential for the development of new therapeutic approaches aimed at both modulating the immune response and restoring the skin barrier, improving more effective clinical management and patients' quality of life.

## Introduction

Atopic dermatitis (AD) is a chronic, relapsing inflammatory skin disease. Skin manifestations include erythema, lichenification, xerosis, papules and desquamation. AD is characterized by intense pruritus, one of the main symptoms, which aggravates others, such as a sensation of pain,^1^ sleep disorders, fatigue^2^, ^3^ and neuropsychiatric symptoms (attention deficit hyperactivity disorder, depression and anxiety).^3^, ^4^ Together, all these manifestations contribute to impairing of patient's quality of life, leading to psychosocial suffering and stigma.^2^

The etiopathogenesis of AD is a complex multifactorial interaction between various elements: environmental factors, genetic susceptibility,^5^ changes in the function of the skin barrier, the microbiota and the immune system.^6^ Together, these factors contribute to the development, progression and chronicity of the disease. As complications, AD patients are more susceptible to infections such as those caused by the *herpes simplex virus* (HSV)^7^ and mainly by *Staphylococcus aureus* (*S. aureus*).^8^, ^9^ Patients with AD are also susceptible to environmental impacts, indicating that climatic factors, temperature, humidity, air pollution,^10^ and exposure to ultraviolet rays^11^ can interact directly with the skin barrier and significantly influence the development and exacerbation of AD symptoms.^12^

The review addresses the relevance of the interaction between alterations in skin barrier proteins, the innate and adaptive immune response, and connections with the inflammasome complex, and the consequent impact on the pheno-endotypical features of AD.

## Key changes in the immune response of atopic dermatitis

### Impaired skin barrier

In AD patients, changes in the skin barrier (SB) lead to increased transepidermal water loss (TEWL), defects in the metabolism of pro-filaggrin,^13^ and lower expression of filaggrin and claudin 1 (protein located in tight junctions ‒ TJ).^14^, ^15^ In addition, there is a decrease in ceramide levels,^13^, ^16^ cholesterol sulfate and accumulation of sphingosylphosphorylcholine, due to increased expression of the enzyme sphingomyelin deacylase.^17^, ^18^ Other contributors include decreased antimicrobial peptides (AMPs), increased serine protease (SP), reduced SP inhibitors and disrupted TJs.^19^

In addition, keratinocytes of AD patients show dysfunction in their response to environmental stimuli, undergoing apoptosis and leading to interruptions in SB function. Also, loss-of-function mutations in the filaggrin gene,^20^ in the claudin 1 protein and single nucleotide polymorphisms in the SP SPINK5 inhibitor and SP KLK7 have been described in AD.^21^, ^22^

**Fig. 1:**
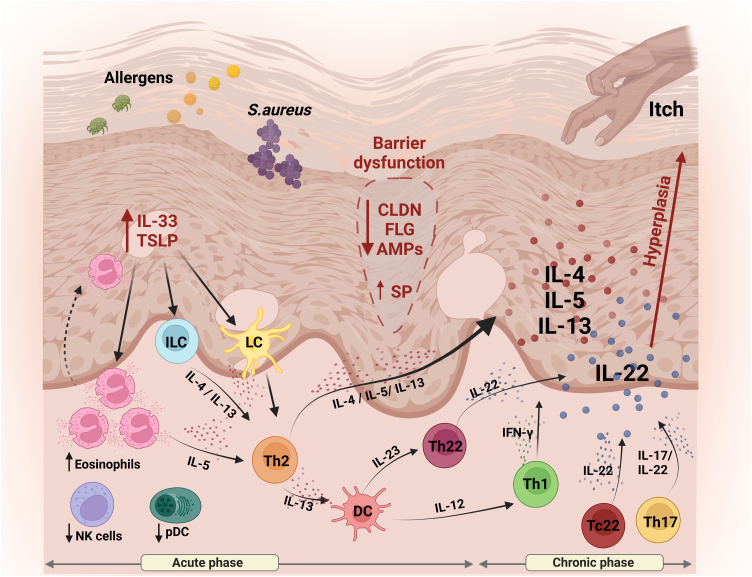
Schematic representation of skin barrier (SC) alterations and the main aspects of innate and adaptive immunity involved in the pathogenesis of AD. Reduced expression of skin proteins (FLG and CLDNs) results in disruption of the BC and transepidermal water loss, facilitating the entry of external pathogens. Keratinocytes release TSLP and IL-33, amplifying the inflammatory response, attracting and activating Langerhans cells, ILCs and the recruitment of eosinophils. Together, these cells contribute to the release of Th2-type cytokines, including IL-4, IL-5 and IL-13. Dendritic cells, in turn, release IL-12 and IL-23, promoting activation of both the Th1 and Th22 profiles, resulting in the secretion of IFN-γ and IL-22, respectively. Pro-inflammatory cytokines IL-5, IL-13 and IL-31 are released by keratinocytes, which play a role in modulating pruritus, contributing to the perpetuation of the inflammatory process. In addition, IL-22 stimulates keratinocyte proliferation, promoting epidermal hyperplasia. IL, Interleukin; TSLP, thymic stromal lymphopoietin; CLDN, claudin; FLG, filaggrin; AMPs, antimicrobial peptides; SP, serine protease; ILC, innate lymphoid cells; LC, Langerhans cells; DC, dendritic cells; NK, natural killer; pDC, plasmacytoid dendritic cells; Th, T-helper. Figure generated with BioRender.

Fragility in the SB is well described as a key factor in the pathogenesis of AD, amplifying inflammatory manifestations and aggravating the symptoms associated with the disease.^23^ The sum of these factors contributes to greater susceptibility to infections and inflammation, due to increased skin permeability to allergens and irritants, favoring the penetration of pathogens (Fig. 1).

### Mechanisms of adaptive immunity

The Th1/Th2 paradigm in AD has been revised, with evidence of a significant role for the IL-17 and IL-22-secreting Th17 and IL-22-secreting Th22 cell subtypes.^24^, ^25^, ^26^, ^27^ Thus, during the acute phase of AD, in addition to Th2 cytokines such as IL-4, IL-13 and IL-31, there is a predominance of IL-22, as well as smaller amounts of Th17 cells.^28^ In the chronic phase of the disease, there is an amplification of Th2 and Th22 cytokine axes, with augmented Th1 cells but no further increases in Th17 cells.^28^ These cytokines reduce the differentiation of epidermal cells and may contribute to the reduction of filaggrin and antimicrobial peptides (AMPs).^29^ Analysis of acute lesions, compared to non-lesioned skin or chronic lesions, showed up-regulation of the S100A7, S100A8 and S100A9 genes and concomitant activation of Th2 and Th22 cytokines.^27^ In addition, there is an increase in IL-22 in the dermis and serum of individuals with AD, suggesting a systemic impact on the immune response (Fig. 1).^30^

The IL-22-producing cells that infiltrate the AD skin lesion are CD4+ T-helper lymphocytes (Th22) and CD8+ T-cytotoxic lymphocytes (Tc22); clinical severity of AD correlated with the number of Tc22 cells, but not with Th22 cells.^31^ In addition, cells from AD patients were responsive to stimulation with staphylococcal enterotoxins, with a decreased response by Th22 cells and increased responsiveness by Tc22 cells, suggesting the role of both T22 cells in the immune imbalance of AD.^30^ Stimulation with enterotoxins also promoted up-regulation of anergy-related genes (EGR2 and IL13) in AD patients, associated with impairment of the effector response of CD4+CD38+ T cells.^32^

As for Th17 cells, their role in defense against bacterial pathogens is well-known and they may be crucial in the pathogenesis of chronic inflammatory skin diseases.^31^ However, there is currently no consensus on the role of these cells in the immunopathogenesis of AD. There is an increase of IL-17 in the skin and serum levels in AD patients.^15^ It is also known that IL-17 exerts a role as an amplifier of skin lesions and the increase in circulating Th17 cells correlates with disease severity.^33^

T-cells positive for cutaneous lymphocyte antigen (CLA+; skin homing receptor) mediate pathogenic inflammation in AD. Circulating CLA+ T cells are elevated in AD, respond to allergens, infiltrate skin lesions and participate in the initiation and perpetuation of AD lesions.^34^ In addition, bacterial toxins are also able to increase the expression of CLA.^35^

In addition to the involvement of cellular immunity, high IgE levels in AD are strongly correlated with the prevalence of IgE autoreactivity and disease severity. More findings reveal an increase of IgG4 and IgE anti-SEB (staphylococcal enterotoxin B) antibodies.^36^ Likewise, numerous infiltrated cells in the skin lesions of individuals with AD are positive for IgE or its high-affinity Fc IgE receptor (FcεRI).^37^

### Mechanisms of innate immunity

In addition to the physical barrier mechanisms previously discussed, epithelial cells, mainly keratinocytes, are crucial components of the skin's innate immunity. These cells behave as sentinels for danger signals or microbial pathogens, triggering immune responses and a cascade of cytokine production.^38^, ^39^

Keratinocytes in AD skin express high levels of thymic stromal lymphopoietin (TSLP), IL-7-like cytokine, which induces the activation and migration to the lymph nodes of dendritic cells (DCs). The DCs stimulated by TSLP induce naive T cells to produce IL-5, IL-13 and TNF-α, and initiate the production of chemokines by the DCs, which attract Th2 cells, an abundant subtype in AD patients (Fig. 1).^38^, ^39^

Epithelial cells and immune cells in the skin barrier express pattern recognition receptors (PRRs) that trigger innate immune responses. The arsenal of PRRs includes members of the Toll-like receptors (TLRs) family, C-type lectin receptors (CLRs), retinoic acid-inducible gene (RIG) cytoplasmic receptors, peptidoglycan recognition proteins (PGLYRPs) and NOD-like receptors (NLRs).^40^, ^41^ The latter will be focused below with more details.

There are reports on the dysfunction of the TLR2, TLR9, and NOD1/2 receptors in AD patients. TLR2 recognizes peptide glycans from gram-positive bacteria and is among the most extensively studied receptors in infectious complications of AD patients. In monocytes and keratinocytes from AD patients, there was impairment of TLR2-mediated inflammatory cytokine production (IL-1β and TNF-α).^42^, ^43^

In addition, the release of AMPs potentiates the strength of TJs and reinforces the defensive barrier against the invasion of microorganisms. In individuals with AD, there is a decrease in the functionality of TLRs, compromising this protective mechanism. This scenario contributes to greater susceptibility to skin infections, especially *S. aureus*.^44^ *S. aureus* virulence factor itself is also an important element of the inflammation in AD patients, capable of inducing TSLP and IL-33, rather than AMPs (Fig. 1).^45^

With regards to cell subtypes in AD, there is evidence of reduced function or migration to the skin of polymorphonuclear effector cells, natural killer (NK) cells and plasmacytoid dendritic cells (pDC).^42^ On the other hand, an increase in circulating eosinophils and eosinophil granule proteins has been reported in the sera and urine of patients.^46^ It is worth noting that during AD crises, there is an increase in circulating IL-5 and eosinophilic chemotaxins, contributing to the extravasation of eosinophils into the skin (Fig. 1).

In AD lesional skin, there is an increased presence of activated NK cells, in contrast to their reduction in peripheral blood.^47^ The authors recently found increased NK CLA + cells in the peripheral blood of patients with severe AD and augmented expression of CD56 and granzyme in the dermis of these individuals. In addition, these cells were responsive to in vitro stimulation with microbial agonists.^48^ There are reports on elevated group 2 Innate lymphoid cells (ILC2), which produce type 2 cytokines (IL-4, IL-5, IL-9 and IL-13) on the lesional skin of AD patients. More findings describe activation of ILCs by TSLP, IL-33 and IL-25, highly expressed in AD.^49^

## Defining inflammasomes and their role in inflammatory skin diseases

Inflammasome is an intracellular protein complex that can be activated in response to pathogens and signs of tissue damage. Its primary function is the maturation and secretion of pro-inflammatory cytokines which are fundamental for the elimination of pathogens and for tissue healing.^50^ As a signaling pathway linked to the innate immune response, the inflammasome cascade is well characterized in myeloid cells, but some skin cells such as keratinocytes express its components.^51^ Despite its crucial role in the innate immune response, deregulated activation of the inflammasome may contribute to the development of inflammatory diseases such as rheumatoid arthritis, autoimmune diseases, inflammatory syndromes and skin diseases.^52^

**Fig. 2:**
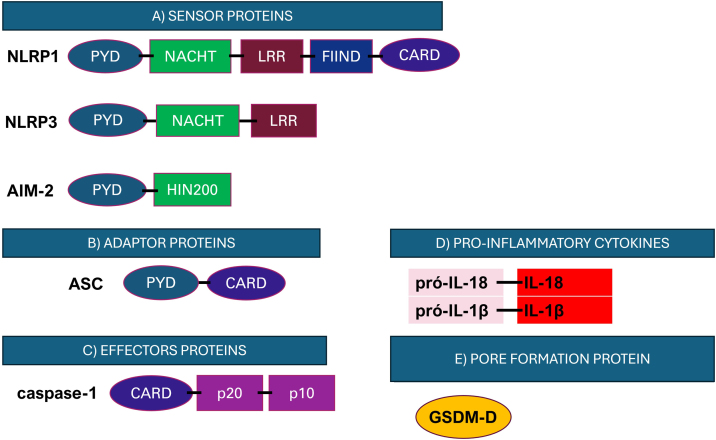
Representation of the structure of the main components of the inflammasome. (A) Initiator proteins comprise cytosolic receptors; (B) ASC adaptor protein; (C) Caspase-1 effector protein; (D) Inflammatory cytokines and (E) GSDM-D pore-forming protein. NLRP, NACHT, LRR and PYD *domains-containing protein*; CARD, caspase recruitment domain; NACHT, Nucleotide-Biding Oligomerization Domain: Nucleotide-Binding Oligomerization Domain; LRR, Leucine-Rich Repeats; PYD, pyrin domain; HIN200, interferon-induced proteins; FIIND, function-to-find domain. Figure generated with BioRender.

The inflammasome signaling cascade begins with the activation of initiator proteins (Fig. 2). These proteins are PRRs that recognize danger signals, such as pathogen-associated molecular patterns (PAMPs) and damage-associated molecular patterns (DAMPs).^53^, ^54^ After activation, the initiator proteins interact with the adaptor protein ASC (apoptosis-associated speck-like protein containing a CARD), which plays an essential role in the assembly of the inflammasome complex. Oligomerization of ASC via its PYD domain promotes the recruitment of pro-caspase-1, acting as a bridge between receptor interactions and pro-caspase-1.^55^ This association results in the autoactivation of the pro-caspase, converting it into the effector protein caspase-1, an inflammatory caspase that cleaves its substrates pro-IL-18, pro-IL-1β into their mature and bioactive forms IL-18 and IL-1β (Fig. 2).^56^, ^57^, ^58^

In addition to the processing and maturation of cytokines, the activation of caspase-1 by the inflammasome cascade may also promote the cleavage of the Gasdermin D (GSDM-D) protein.^59^ GSDM-D was first described in 2015 and is expressed in immune cells and epithelial cells. After cleavage, the N-terminal portion of GSDM-D is released and inserts itself into the cell membrane, resulting in the formation of pores in the plasma membrane that lead to the release of the cytokines IL-1β and IL-18, and a highly inflammatory cell death process called pyroptosis.^55^, ^57^, ^60^, ^61^, ^62^

**Fig. 3:**
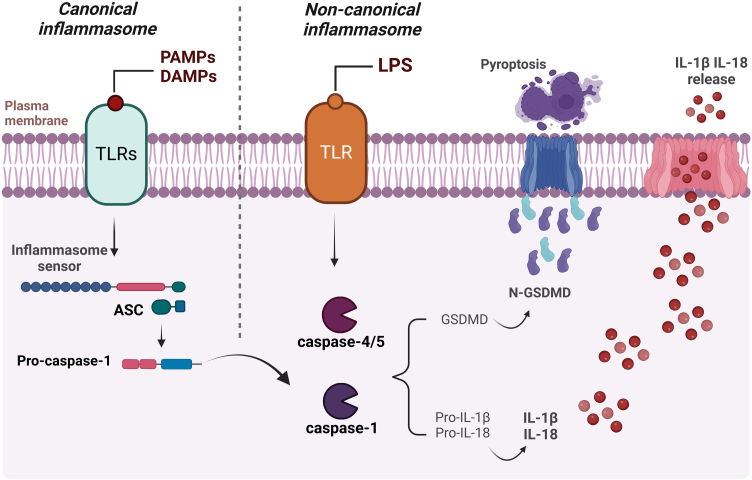
Schematic representation of canonical and non-canonical inflammasome activation. The formation of the inflammasome via the canonical pathway occurs when PAMPs, DAMPs or other cytosolic disturbances are detected, resulting in the recruitment and activation of caspase-1, either directly or through the recruitment of the adaptor protein ASC. Caspase-1 initiates the process of maturation of pro-IL-1β and pro-IL-18 into their active forms, in addition to cleaving GSDM-D. This, in turn, interacts with the plasma membrane, creating pores and resulting in the release of intracellular content, including the inflammatory cytokines IL-1β and IL-18. Activation of the non-canonical inflammasome begins with the detection of cytosolic LPS by caspase-4/caspase-5 (or pro-caspase-11 in mice), triggering the cleavage of GSDM-D and subsequent pyroptosis. Figure generated with BioRender.

In addition to the canonical activation of the inflammasome mentioned above, the cytokines IL-18 and IL-1β can also be produced through non-canonical activation of the inflammasome, when lipopolysaccharide (LPS) is directly recognized by the inflammatory caspases -4 and -5 in humans and caspase-11 in mice.^63^, ^64^ The main activation mechanisms of the canonical and non-canonical pathways are presented in Fig. 3.

Among the inflammasome receptors, the family of NOD-type receptors is the best characterized.^65^ These receptors have a tripartite structure composed of a central nucleotide-binding and oligomerization domain (NACHT), usually accompanied by leucine-rich repeats (LRRs), a C-terminal domain that recognizes and regulates ligands, and a variable N-terminal domain, which can be a caspase recruitment domain (CARD) or pyrin (PYD), responsible for the effector functions of the complex.^65^ The NLR family has three distinct subfamilies: NODs (NOD1-2), NLRPs (NLRP1-14), and IPAF (ice protease-activating factor).^65^

The NLRP1 inflammasome was first described in 2001 through the identification of the N-terminal PYD domain by Martinon et al.^64^ The authors demonstrated the interaction of NLRP-1 with caspase-1 dependent on the presence of the ASC protein constituting the inflammasome complex.^64^ NLRP1 is expressed in various immune cells, mainly in the skin by keratinocytes, and appears to play a central role as the main receptor involved in the formation of the inflammasome.^66^ Indeed, UVB irradiation in primary keratinocytes causes translocation of the ASC protein to the nucleus with the formation of aggregates known as speck and this complexation is dependent on NLRP1.^67^ NLRP1 also recognizes the main virulence factor of *Bacillus anthracis* and bacterial muramyl dipeptide.^68^ In humans, gain-of-function mutations in the NLRP1 gene lead to syndromes mediated by inflammasome activation in keratinocytes characterized by skin inflammation and susceptibility to skin cancer.^69^

The most well-known inflammasome is NLRP3, which is expressed by epithelial cells, macrophages, lymphoid cells, chondrocytes and skin keratinocytes.^70^ This receptor can be activated by a range of PAMPs and DAMPs that do not interact directly with the receptor, but induce cytoplasmic changes that lead to its activation, such as ion flows, extracellular ATP, nucleic acids, bacterial toxins, among others, and is involved in various skin diseases.^71^, ^72^, ^73^, ^74^, ^75^ In keratinocytes, UVB radiation triggers NLRP3 in increased concentrations of cytoplasmic calcium (Ca^2+^), leading to the secretion of IL-1β.^66^, ^76^

In patients with progressing vitiligo, NLRP3 and IL-1β concentrations are increased in perilesional epidermal samples when compared to healthy controls.^77^ In psoriasis there is higher expression of NLRP3 and of several inflammasome components such as caspase-1 and IL-1β.^78^, ^79^ A close relationship has been described between the pathogenesis of leprosy, colorectal cancer, rheumatoid arthritis, abdominal aortic aneurysms, inflammatory bowel disease, ulcerative colitis and AD, and the NLRP3 rs35829419 gene polymorphism.^80^, ^81^ Bacteria (*S. aureus* and *Escherichia coli* e.g.) and environmental exposure to ultraviolet radiation led to the activation of the NLRP3 inflammasome in keratinocytes.^71^, ^82^ NLRP3 is also necessary for the pyroptotic cell death of macrophages infected with *S. aureus*.^74^

AIM-2 is a receptor first described as a tumor cell growth suppressor gene in melanoma that does not belong to the NLR family, but is capable of forming the inflammasome complex after binding directly to dsDNA (double-stranded DNA).^83^, ^84^ Under homeostasis, DNA remains contained in the nucleus and mitochondria, but exposure of DNA in the cytosol indicates active infection or cell damage. The AIM2 receptor responds to the cytosolic presence of the host's own DNA and amplifies sterile inflammation, as described in autoinflammatory or autoimmune diseases.^85^ Furthermore, AIM2 also recognizes exogenous DNA released during bacterial infections, such as *S. aureus*,^86^ or viral infections such as cytomegalovirus (CMV), and also upon detection of HPV-16 in keratinocytes, triggering an increase in the secretion of IL-1β.^72^, ^87^, ^88^

In psoriasis, cytosolic dsDNA also stimulates the activation of AIM2 expression and IL-1β secretion. Interestingly, LL-37 inhibits the ability of DNA to induce IL-1β production due to LL-37's strong association with DNA, which prevents it from becoming involved in the AIM-2 inflammasome and contributing to the pathophysiology of the disease.^89^ In patients with lupus erythematosus, an intense expression of AIM-2 was observed in macrophages, possibly due to decreased DNA methylation in these individuals, contributing to the pathogenesis of the disease.^90^, ^91^ Moreover, in lesions from patients with lichen planus, there is a specific increase in the AIM-2 protein in both the dermis and the epidermis, with improved dermal expression of the IL-1β protein.^92^ Based on these findings, AIM-2 may be a therapeutic target in inflammatory and oncologic conditions, and also in autoimmune diseases.^93^, ^94^

## Inflammasomes and atopic dermatitis

Considering the inflammatory profile of AD, the inflammasome cascade may exert a relevant role in the pathogenesis of the disease. In fact, the activation of inflammasomes by allergens or pathogens leads to increased levels of IL-1β on the skin in autoinflammatory diseases, promoting conditions for the development of chronic inflammation.^95^

Polymorphisms and mutations in the NOD1 and NOD2 genes are associated with high levels of IgE in AD patients and are relevant factors indicating susceptibility to atopy.^96^, ^97^ Similarly, polymorphisms in the NLRC4 gene, which also codes for an NLR, are associated with AD;^97^ however, functional studies on the role of this receptor in the pathogenesis of the disease are still scarce.

In addition to polymorphisms, the skin of AD patients shows an increase in inflammasome components, which may favor inflammation. In fact, the highest expression of AIM-2 was found in keratinocytes from AD patients and is directly associated with acute and chronic inflammation related to SB rupture.^98^, ^99^ These patients have intense pruritus, and the mechanical act of scratching impairs SB, causing intracellular products to be released and detected by the AIM-2 receptor. Moreover, bacterial DNA can be released after a process of bacteriolysis mediated by AMPs, and keratinocytes are able to capture exogenous DNA through receptor-mediated endocytosis.^86^

The expression of NLRP1 and NLRP3 receptors is also increased in the damaged skin of AD patients and is directly associated with the severity of the disease, highlighting the importance of this signaling cascade for the inflammatory pathology of this and other skin diseases.^70^, ^98^, ^100^ In 2010, Grigoryev et al.,^100^ showed that NLRP1 gene expression is inversely correlated with AD severity in skin explants, suggesting that local inflammation could inhibit NLRP1 expression, or that reduced expression of this protein promotes skin inflammation. Furthermore, higher NLRP1 expression and caspase-1 activity were seen in mild AD patients and is associated with IL-1β and IL-18 production.^101^ Several studies have described the relevance of variations in the NLRP1 gene in skin diseases such as vitiligo, psoriasis and leprosy, showing that NLRP1 plays a particular role in the skin.^102^, ^103^, ^104^

Considering NLRP3, the expression of this receptor is directly associated with an increase in IL-33 in the lesions of AD patients, but this association is independent of the activation of the inflammasome cascade.^70^ In addition, Cho et al.^105^ demonstrated that IL-17 and IL-22 secreted by Th17 cells can activate NLRP3 and stimulate the secretion of IL-1β and caspase-1 in immortalized HaCaT keratinocytes, suggesting that other pathways such as the TH17/Th22 axis play a role in the activation of the inflammasome complex. In vitro tests showed that inhibition of Drp1 (dynamin-related protein-1) ‒ responsible for NLRP3 activation ‒ with the compound mdivi-1 inhibited NLRP3 activation, IL1β and IL-18 production, as well as induction of pyroptosis.^106^ This compound was also shown to be effective in improving the symptoms associated with AD, as well as reducing the serum IgE levels and the production of IL-4, IL-5 and IL-13 in skin lesions in a murine model of AD.^106^ In addition, omega-3 supplementation decreases NLRP3 activation via NF-κB, and decreases the expression of Th2 cytokines in experimental mouse models of AD, suggesting that this receptor is a target for therapeutic strategies for the disease.^107^

Conversely, Niebuhr et al.^108^ showed reduced expression of NLRP3 and caspase-1 in AD skin. In addition, a reduction in NLRP3 and ASC transcripts was observed in keratinocytes stimulated with Th2 cytokines (IL-4, IL-5 and IL-13).^108^ These cytokines can also reduce caspase-1-dependent IL-1β secretion in monocytes from AD patients stimulated with staphylococcal α-toxin, suggesting interference of the NLRP3 inflammasome in the Th2 response profile, which is relevant in the pathogenesis of AD.^108^ The controversial findings may be the result of differences between animal and human studies, with great variability in the number of participants, indicating the need for further analysis to better understand the role of NLRP3 in the AD inflammatory response.

Recently, Ramos et al.^98^ showed that there is an alteration of other components of the inflammasome beyond NLRP3 and NLRP1 receptors in AD. The authors found that ASC expression by immunohistochemistry is increased in the dermis of AD patients, as well as caspase-1.^98^ Furthermore, human primary keratinocytes show enhanced ASC expression when stimulated with UV.^67^

Ramos et al.^98^ also identified that individuals with AD have a higher expression of GSDM-D, which may favor inflammation and local tissue damage. Interestingly, in a murine experimental model of AD induced by oxazolone, the inhibition/deletion of the GSDM-D gene decreased IL-1β and IL-18 levels, confirming the involvement of pyroptosis in amplifying the inflammatory response in AD.^109^ In this regard, the GSDM-D deletion promotes an improvement in AD-like lesions by decreasing the cellular infiltrate and reducing IgE and IL-4 levels.^109^ GSDM-D is also elevated in psoriatic skin lesions, but serum levels of this protein are similar to the healthy control group.^110^

In counterpart, the relationship between the inflammasome cascade and colonizing bacteria in the skin microbiota of AD patients has also been studied. Recent evidence indicates that several inflammasomes are activated during an *S. aureus* infection, including NLRP3, via NF-κB, increasing the transcription of pro-IL-1β, activation of caspase-1, and secretion of IL-1β, together with IL-18.^111^ In macrophages, *S. aureus* γ-hemolysins can activate the NLRP3 inflammasome and caspase-1 without the participation of the P2 × 7 receptor or the Myd88/TLR adaptor. In addition, inoculation of *S. aureus* into keratinocytes led to increased secretion of IL-1β and IL-18, and silencing of NLRP1 inhibited the production of these cytokines, suggesting that skin colonization may lead to activation of this receptor directly interfering with the inflammatory response in the disease.^112^

In skin diseases associated with dysregulation of the inflammasome, the effector cytokines IL-1β and IL-18 are highly expressed, playing a significant role in the onset and exacerbation of inflammation.^113^ In fact, cutaneous keratinocytes are the main sources of these cytokines in the skin, and the increased expression of inflammasome components associated with exposure to *S. aureus* antigens may lead to an increase in IL-18 and IL-1β in AD patients.^66^, ^114^ In fact, CD68+ macrophages produce IL-1β in the dermis of AD patients,^98^ and increased epidermal expression of this cytokine is observed in AD patients with filaggrin gene mutations.^115^ Interestingly, several observations suggest that both IL-1α and IL-1β contribute to the development of skin inflammation with AD.^116^ Additionally, mast cells and keratinocytes from AD patients produce IL-18 in response to exposure to allergens or pathogens such as dust mites and *S. aureus*.^117^ The IL-18 produced stimulates basophils, mast cells, and CD4 T-cells to produce Th2 cytokines in acute AD lesions, while in chronic lesions, IL-18 stimulates Th1 cells to produce IFN-γ together with IL-12.^118^

The inflammatory profile triggered by inflammasome activation is also systemically reflected. Orfali et al.^114^ showed high concentrations of IL-18 in the serum of AD patients according to the severity of the disease, regardless of the presence of staphylococcal enterotoxins. IL-18 is also increased in the culture supernatants of mononuclear cells from patients stimulated with staphylococcal enterotoxin type A (SEA).^114^ However, studies on IL-18 deficient mice show an absence of this cytokine, leading to a reduction in the worsening of skin lesions.^119^ The main changes in inflammasomes related to the pheno-endotypical features in AD are summarized in Table 1.

**Table 1:** Association of the inflammasome with pheno-endotypical features of AD.

Inflammasome component	Main findings	References
NOD1 e NOD2	NOD1 and NOD2 gene polymorphisms associated with high IgE levels and susceptibility to atopy	96, 97
NLRP1	NLRP1 expression inversely correlated with AD severity	100
NLRP3	NLRP3 gene polymorphism associated with the development of AD	80, 81
	γ-hemolysin from *S. aureus* leads to NLRP3 activation	112
	Reduced expression of NLRP3 and caspase-1 in AD patients, relationship with Th2 response profile	108
NLRC4	NLRC4 gene polymorphisms associated with AD	97
AIM-2	Increased expression of AIM-2 in keratinocytes, associated with inflammation and breakdown of the skin barrier	98, 99
ASC Caspase-1	Increased expression of ASC and caspase-1 in lesions of AD patients, associated with inflammation	67, 98
GSDM-D	GSDM-D deletion in a murine model reduces IL-1β, IL-18 and AD symptoms, indicating involvement in pyroptosis	109
IL-18	Elevated IL-18 levels in serum and peripheral blood mononuclear cells of AD patients	114

**Fig. 4:**
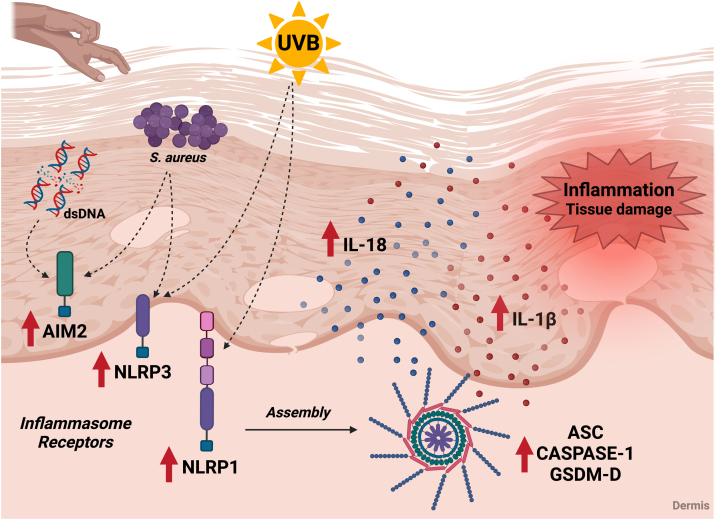
Association of the inflammasome cascade with AD immunopathology. Components of the inflammasome signaling pathway are increased in the skin of AD patients. In the skin of patients, inflammasome receptors can be activated by exposure to ultraviolet radiation, by contact with *S. aureus* antigens, and by possible DNA molecules released by cell damage. These receptors promote the oligomerization of ASC and caspase-1, leading to the maturation of IL-1β and IL-18, which are also elevated in AD patients. In addition, activation of the inflammasome leads to an increase in GSDM-D and promotion of pyroptosis. The release of pro-inflammatory and cell death cytokines contributes to tissue damage and local and systemic inflammation during the disease. UVB, ultraviolet radiation; *S. aureus*, *Staphylococcus aureus*; dsDNA, double stranded DNA. Figure generated with BioRender.

The above findings point out the relevance of inflammasome components in AD as potential biomarkers of the disease and possible targets for future immunomodulatory interventions (Fig. 4).

## Targeted therapies and relevance of individualized therapy for key changes in AD

The use of emollients and topical corticosteroids is the first line of treatment for AD.^120^ Conventional systemic therapy options for AD include cyclosporine (CsA), methotrexate, azathioprine, mycophenolate mofetil, and systemic glucocorticoids, with limited proven efficacy and potential side effects.^121^ With the development of new target-specific therapies aimed at inflammatory mediators, new opportunities have arisen for restoring the skin barrier, as well as for restoring the innate and adaptive immune systems in AD.^121^

One of the target therapies is dupilumab (DUPI), a humanized monoclonal antibody that targets IL-4Rα, a subunit shared by the IL-4 and IL-13 receptors. Pivotal studies have shown significant clinical improvement and a favorable safety profile in individuals with moderate to severe AD, confirming the central role of Th2 cytokines in this disease.^121^ It was shown that blocking IL-4/IL-13 signaling, in addition to suppressing systemic Th2-type inflammation, increased microbial diversity, reducing the abundance of *S. aureus*, with the recovery of the altered transcriptome of epidermal proteins associated with AD. DUPI also increased the expression of FLG, LEKTI (protease inhibitor), and HBD-3 (antimicrobial peptide) after 6‒8 weeks of treatment, as well as the degree of hydration of the stratum corneum, with a consequent improvement in the severity of AD after 12 weeks. Transcriptomic data from the European clinical registry (TREAT Germany) of adults with moderate to severe AD, in a comparative study of the use of DUPI vs. CsA, showed that treatment with DUPI for 12 weeks led to the normalization of BC-related genes, to a greater extent than CsA,^122^, ^123^ and decreased expression of chemokines related to the Th2 response (e.g. CCL13, CCL17, CCL18 and CCL22). There was normalization of the expression of markers related to barrier function (e.g., CLDN8, ELOVL3, FLG, K1, K10 e LOR).^19^, ^124^, ^125^, ^126^

Other IL-13 antagonists (lebriquizumab and tralokinumab) have shown clinical efficacy in AD patients, demonstrating the critical role of IL-13 in the pathogenesis of AD. Since IL-4 and IL-13 reduce the expression of CB proteins such as filaggrin, loricrin, and involucrin, treatment with IL-13 antagonists is expected to positively influence the recovery of CB integrity, but more studies are needed to corroborate these findings.^19^

Janus Kinase (JAK) inhibitors that target JAK/STAT signaling, such as baricitinib, upadacitinib and abrocitinib, have shown promising results in some studies regarding the restoration of SB, demonstrating an increase in filaggrin expression, as well as a reduction in inflammatory signaling.^19^ There is a lack of publications focusing on the role of JAK inhibitors and new immunobiologicals in the recovery of SB and its components, especially targeting elements of the inflammatory response such as OX-40 Ligand (OX-40 L) and OX-40 (amlitelimab and rocatinlimab),^127^ as well as IL-31 (nemolizumab).^128^

Regarding the therapeutic potential of inhibiting inflammasomes, NLRP3 inhibitors and their respective mechanisms in allergic diseases have been described. These include MCC950, a diarylsulfonylurea-based compound that inhibits NLRP3 activity and interferes with chloride efflux; OLT1177, an orally active b-sulfonyl cyanide molecule that binds directly to NLRP3 and inhibits ATPase activity, preventing NLRP3-ASC, NLRP3-caspase-1 interaction; CY-09, an inhibitor of cystic fibrosis transmembrane conductance regulator (CFTR) channels that inhibits NLRP3 ATPase activity; Tranilast, a tryptophan metabolite that inhibits NLRP3 oligomerization and improves NLRP3 ubiquitination; Oridonin (ent-kaurane diterpene), the main active component of *Rabdosia rubescens* that blocks the interaction between NLRP3 and NEK7; RRx-001, a pleiotropic anticancer agent that blocks the interaction between NLRP3 and NEK7. Natural products and their derivatives are also suggested as potential therapeutic strategies such as XQLD (*Xiaoqinglong Decoction*) which inhibits NLRP3 inflammasome-mediated pyroptosis; APS (*Astragalus* Polysaccharide), an inhibitor of NLRP3 activation and a blocker of NF-kB phosphorylation, decreasing NOD2 expression; MFXD (*Mahuang Fuzi Xixin Decoction*), which inhibits the NLRP3/Caspase-1/GSDMD-N signaling pathway; *Schisandrin B*, which inhibits NLRP3 activation; *Houttuynia cordata*, which decreases the expression of NLRP3, ASC, caspase-1, GSDMD, IL-1β, and IL-18; and *Angelica Yinzi*, which inhibits NLRP3 activation and MAPKs/NFkB signaling.^129^, ^130^ Currently, there is a clinical trial for AD patients with GSK1070806, an anti-IL-18 monoclonal antibody. In phase 1b of the study NCT04975438 (https://clinicaltrials.gov/study/NCT04975438?tab=history&a=7#study-results-card), patients with no previous systemic treatment with biologics (non-responsive to topical therapies), and patients non-responsive or intolerant to DUPI were evaluated, with promising results. An ongoing phase 2b will evaluate the clinical effect, safety and tolerability of GSK1070806 in AD (NCT05999799 - https://clinicaltrials.gov/study/NCT05999799?intr=GSK1070806&rank=2).

## Conclusion

AD is one of the most common chronic inflammatory skin diseases, and its persistence occurs as a consequence of the combination of genetic, environmental and immunological factors, affecting both adults and children. Skin barrier dysfunction, together with bacterial colonization and dysregulation of the innate and adaptive immune system, play a central role in maintaining inflammation and therefore the chronicity of AD. This review highlights the crucial role of inflammasomes, especially NLRP1/3 and AIM-2, in regulating the inflammatory response in skin diseases such as AD, including the release of the pro-inflammatory cytokines IL-18 and IL-1β, which are essential in perpetuating skin inflammation. A deeper understanding of immune pathways, such as the inflammasome cascade, opens new perspectives for therapeutic interventions. Targeting treatments to modulate the immune response and restore the skin barrier may offer more effective management of the disease and improve the AD patients' quality of life.

## Author's contribution

Yasmim Álefe Leuzzi Ramos: Critical review of the literature; drafting of the manuscript; preparation of figures; final approval of the final version of the manuscript.

Anna Julia Pietrobon: Critical review of the literature; drafting of the manuscript; preparation of figures; final approval of the final version of the manuscript.

Franciane Mouradian Emidio Teixeira: Critical review of the literature; drafting of the manuscript; preparation of figures; final approval of the final version of the manuscript.

Valeria Aoki: Conception and design of the study; critical review of the content; analysis and interpretation of the data; critical review of the literature; final approval of the final version of the manuscript.

Maria Notomi Sato: Conception and design of the study; critical review of the content; analysis and interpretation of the data; critical review of the literature; final approval of the final version of the manuscript.

Raquel Leão Orfali: Conception and design of the study; data collection; writing of the article and critical review of the content; obtaining, analyzing and interpreting the data; critical review of the literature; final approval of the final version of the manuscript.

## Financial support

None declared.

## Conflicts of interest

None declared.
